# Preoperative chronic kidney disease predicts poor oncological outcomes after radical nephroureterectomy in patients with upper urinary tract urothelial carcinoma

**DOI:** 10.18632/oncotarget.20554

**Published:** 2017-08-24

**Authors:** Hirotake Kodama, Shingo Hatakeyama, Naoki Fujita, Hiromichi Iwamura, Go Anan, Ken Fukushi, Takuma Narita, Toshikazu Tanaka, Yuka Kubota, Hirotaka Horiguchi, Masaki Momota, Koichi Kido, Teppei Matsumoto, Osamu Soma, Itsuto Hamano, Hayato Yamamoto, Yuki Tobisawa, Tohru Yoneyama, Takahiro Yoneyama, Yasuhiro Hashimoto, Takuya Koie, Hiroyuki Ito, Kazuaki Yoshikawa, Atsushi Sasaki, Toshiaki Kawaguchi, Makoto Sato, Chikara Ohyama

**Affiliations:** ^1^ Department of Urology, Hirosaki University Graduate School of Medicine, Hirosaki, Japan; ^2^ Department of Urology, Tohoku Medical and Pharmaceutical University, Sendai, Japan; ^3^ Department of Advanced Transplant and Regenerative Medicine, Hirosaki University Graduate School of Medicine, Hirosaki, Japan; ^4^ Department of Urology, Aomori Rosai Hospital, Hachinohe, Japan; ^5^ Department of Urology, Mutsu General Hospital, Mutsu, Japan; ^6^ Department of Urology, Tsugaru General Hospital, Goshogawara, Japan; ^7^ Department of Urology, Aomori Prefectural Central Hospital, Aomori, Japan

**Keywords:** upper urinary tract urothelial carcinoma, radical nephroureterectomy, chronic kidney disease, renal function, oncological outcome

## Abstract

**Objective:**

To evaluate the impact of preoperative chronic kidney disease (CKD) on oncological outcomes in patients with upper tract urothelial carcinoma who underwent radical nephroureterectomy.

**Methods:**

A total of 426 patients who underwent radical nephroureterectomy at five medical centers between February 1995 and February 2017 were retrospectively examined. Oncological outcomes, including intravesical recurrence-free, visceral recurrence-free, cancer-specific, and overall survival rates (intravesical RFS, visceral RFS, CSS, and OS, respectively) stratified by preoperative CKD status (CKD vs. non-CKD) were investigated. Cox proportional hazards regression analysis was performed using inverse probability of treatment weighting (IPTW) to evaluate the impact of preoperative CKD on prognosis and a prognostic factor-based risk stratification nomogram was developed.

**Results:**

Of the 426 patients, 250 (59%) were diagnosed with CKD before radical nephroureterectomy. Before the background adjustment, intravesical RFS, visceral RFS, CSS, and OS after radical nephroureterectomy were significantly shorter in the CKD group than in the non-CKD group. Background-adjusted IPTW analysis demonstrated that preoperative CKD was significantly associated with poor visceral RFS, CSS, and OS after radical nephroureterectomy. Intravesical RFS was not significantly associated with preoperative CKD. The nomogram for predicting 5-year visceral RFS and CSS probability demonstrated a significant correlation with actual visceral RFS and CSS (*c*-index = 0.85 and 0.83, respectively).

**Conclusions:**

Upper tract urothelial carcinoma patients with preoperative CKD had a significantly lower survival probability than those without CKD.

## INTRODUCTION

Upper tract urothelial carcinoma (UTUC) is a relatively rare and heterogeneous disease that accounts for approximately 5% of all urothelial tumors [1]. Radical nephroureterectomy (RNU) with bladder cuff excision remains the standard treatment modality for UTUC [2]. However, the prognosis of patients with advanced UTUC has not improved over the past two decades [1, 3–5]. Established predictors of prognosis in patients with UTUC have been reported, including older age, tumor stage, presence of hydronephrosis, tumor location, lymphovascular invasion (LVI), lymph node involvement [3, 6]. Recent evidence has suggested that preoperative renal insufficiency indicates poor prognosis in urothelial carcinoma including bladder cancer [7–10]. However, the impact of preoperative renal impairment on prognosis remains unclear. Although chronic kidney disease (CKD) is common in elderly patients with UTUC [1, 4–6], few studies have evaluated the direct influence of preoperative CKD on oncological prognosis in UTUC patients after RNU [10, 11]. In the present study, we compared oncological outcomes between UTUC patients with and without preoperative CKD using inverse probability of treatment weighting (IPTW) via a propensity score and developed a prognostic factor-based risk stratification nomogram.

## RESULTS

### Baseline characteristics

Of the 426 patients, 250 (59%) were diagnosed with CKD (estimated glomerular filtration rate [eGFR] <60 mL/min/1.73 m^2^) before RNU. There were significant differences in patient characteristics between the groups in terms of age (*P* < 0.001), smoking status (*P* = 0.009), preoperative eGFR (*P* < 0.001), presence of preoperative hydronephrosis (*P* < 0.001), ≥cT3 (*P* = 0.007), tumor location (*P* = 0.009), laparoscopic surgery (*P* = 0.024), ≥pT3 (*P* = 0.003), pN+ (*P* = 0.022), and LVI+ (*P* = 0.001). There were no significant differences in the number of patients with mixed histology (*P* = 0.472), and carcinoma *in situ* (CIS) (*P* = 0.082) between the groups. The number of patients with adjuvant/salvage chemotherapy after RNU were significantly higher in the CKD group (n = 47, 19%) than those of the non-CKD group (n = 20, 11%) (*P* = 0.038) (Table 1).

**Table 1:** Background of patients

	All	CKD	non-CKD	*P value*
n	426	250	176	
Age (years)	70 ± 8.9	72 ± 8.4	68 ± 8.9	<0.001
Gender (Male), n=	290 (68%)	170 (68%)	120 (68%)	0.968
ECOG-PS > 1, n=	10 (2.3%)	5 (4.0%)	5 (5.7%)	0.755
Hypertension, n=	185 (43%)	113 (45%)	72 (41%)	0.379
Diabetes Mellitus, n=	70 (16%)	40 (16%)	30 (17%)	0.776
Cardiovascular disease, n=	75 (18%)	47 (19%)	28 (16%)	0.437
Smoking, n=	193 (45%)	100 (40%)	93 (53%)	0.009
eGFR before surgery (mL/min/1.73m^2^)	58 ± 18	46 ± 9.6	75 ± 14	<0.001
Hydronephrosis, n=	266 (62%)	183 (73%)	83 (47%)	<0.001
Neoadjuvant chemotherapy, n=	102 (24%)	65 (26%)	37 (21%)	0.231
≥cT3, n=	229 (54%)	148 (59%)	81 (46%)	0.007
cN+, n=	34 (8.0%)	25 (10%)	9 (5.1%)	0.077
Tumor location, n=				
Renal pelvis	166	83	83	
Ureter	235	153	82	0.009
Multiple	25	14	11	
Laparoscopic surgery, n=	75 (18%)	35 (14%)	40 (23%)	0.024
Postoperative complications (Grade ≥ 3), n=	14 (3.3%)	9 (3.6%)	5 (2.8%)	0.665
≥pT3, n=	182 (43%)	122 (49%)	60 (34%)	0.003
Mixed histology, n=	16 (3.8%)	8 (3.2%)	8 (4.5%)	0.472
SCC contained, n=	12 (2.8%)	6 (2.4%)	6 (3.4%)	
AC contained, n=	2 (0.47%)	0 (0%)	2 (1.1%)	
Others, n=	2 (0.47%)	2 (0.8%)	0 (0%)	
Presence of CIS, n=	22 (5.2%)	9 (3.6)	13 (7.4%)	0.082
pN+, n=	30 (7.0%)	24 (9.6%)	6 (3.4%)	0.022
High grade, n=	397 (93%)	235 (94%)	162 (92%)	0.326
Surgical margin positive, n=	14 (3.3%)	11 (9.4%)	3 (1.7%)	0.169
Lymphovascular invasion, n=	127 (30%)	90 (36%)	37 (21%)	0.001
Median follow-up (Months)	40	36	43	
Disease recurrence, n=				
Intravesical	113 (27%)	75 (30%)	38 (22%)	0.053
Visceral	109 (26%)	88 (35%)	21 (12%)	<0.001
Chemotherapy after RNU, n=	67 (16%)	47 (19%)	20 (11%)	0.038
Adjuvant therapy	9 (2.1%)	6 (2.4%)	3 (1.7%)	0.741
Salvage therapy	58 (14%)	41 (16%)	17 (9.7%)	0.046
Survival, n=				
Cancer-specific mortality	80 (19%)	64 (26%)	16 (9.1%)	<0.001
Overall mortality	103 (24%)	78 (31%)	25 (14%)	<0.001

UC: urothelial carcinoma, SCC: squamous cell carcinoma, AC: adenocarcinoma, CIS: carcinoma *in situ*, RNU: radical nephroureterectomy.

### Oncological outcomes

**Figure 1:**
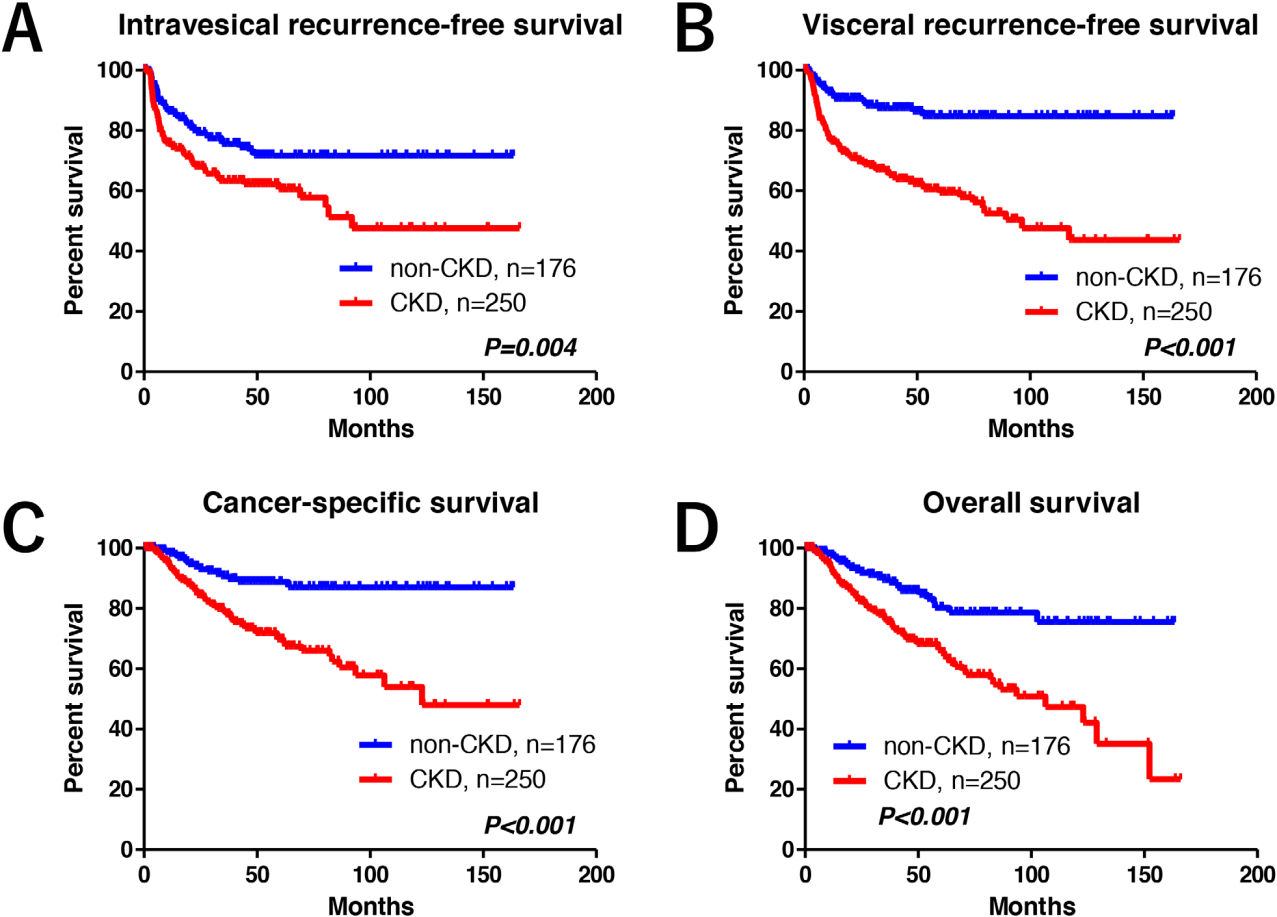
Oncological outcomes Before the background adjustment, statistically significant differences were observed in intravesical recurrence-free **(A)**, visceral recurrence-free **(B)**, cancer-specific **(C)**, and overall survival **(D)** between the groups.

Overall, 181 patients (43%) experienced disease recurrence after RNU. The number of patients with intravesical and visceral recurrences in the median follow-up of 40 months were 113 (27%) and 109 (26%), respectively. Before the background adjustment, there were significant differences in the number of patients experiencing visceral recurrence (*P* < 0.001), cancer mortality (*P* < 0.001), and overall mortality (*P* < 0.001) in the CKD group compared with the non-CKD group (Table 1). There was no significant difference in intravesical recurrence between the groups (*P* = 0.053). The median follow-up periods in the CKD and non-CKD groups were 36 and 43 months, respectively. There were statistically significant differences in intravesical recurrence-free, visceral recurrence-free, cancer-specific, and overall survival (intravesical RFS, visceral RFS, CSS, and OS, respectively) rates between the groups (Figure 1A–1D). The CKD group had significantly worse oncological outcomes than the non-CKD group. Five-year intravesical RFS, visceral RFS, CSS, and OS rates for the CKD and non-CKD groups were 60% vs. 72% (Figure 1A, *P* = 0.004), 59% vs. 85% (Figure 1B, *P* < 0.001), 70% vs. 89% (Figure 1C, *P* < 0.001), and 66% vs. 80% (Figure 1D, *P* < 0.001), respectively.

**Figure 2:**
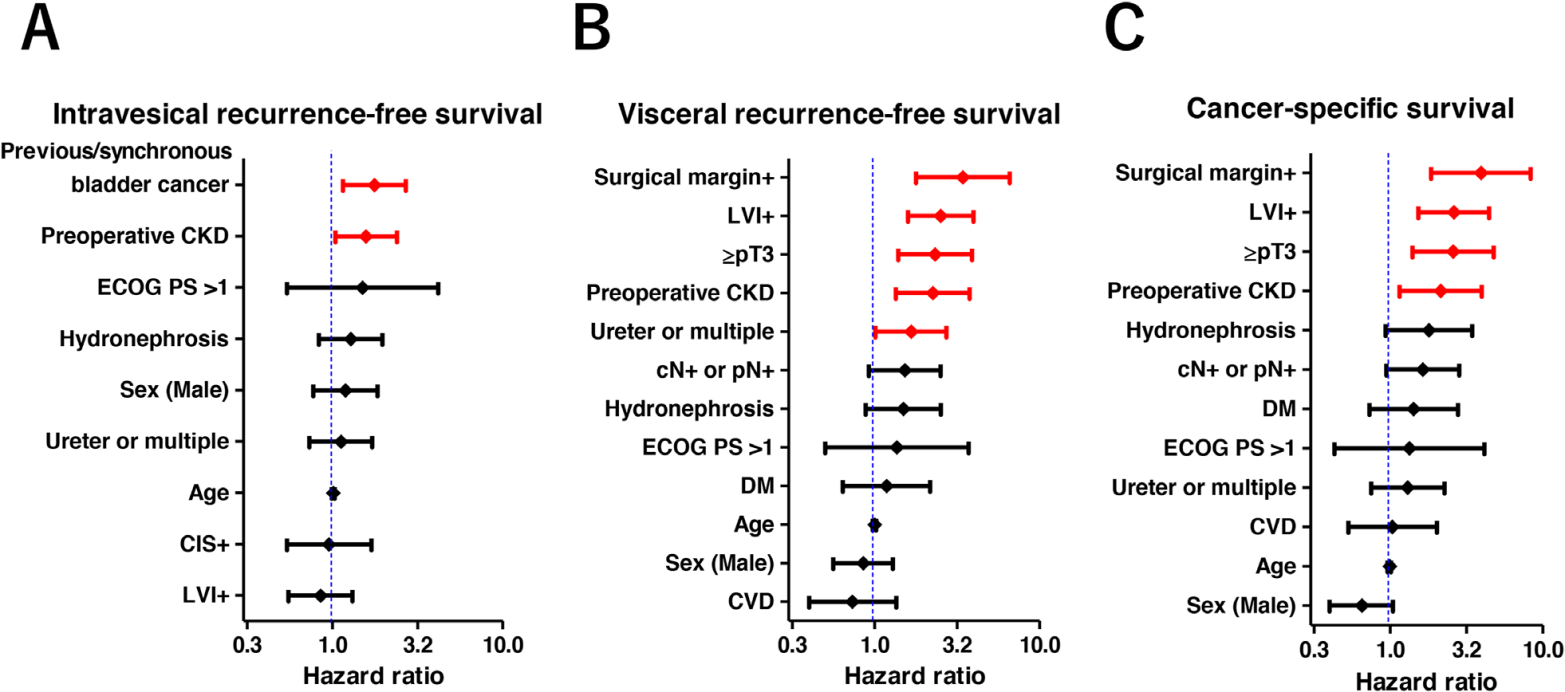
Multivariate Cox regression models for intravesical recurrence-free, visceral recurrence-free, and cancer-specific survival Previous/synchronous bladder cancer and preoperative CKD were identified as independent factors for intravesical recurrence-free survival **(A).** Surgical margin, lymphovascular invasion (LVI), pathological T3 or higher, preoperative CKD, and ureter or multiple tumors were identified as independent factors for visceral recurrence-free survival **(B).** Similarly, surgical margin, LVI, pathological T3 or higher, and preoperative CKD were identified as independent factors for cancer-specific survival **(C)**.

In the multivariate Cox proportional hazards regression analysis, previous/synchronous bladder cancer and preoperative CKD were determined as independent predictors of intravesical RFS (Figure 2A). Similarly, surgical margin, LVI, ≥pT3, preoperative CKD, and tumor location (ureter or multiple) were determined as independent predictors of visceral RFS (Figure 2B). Independent predictors of CSS were surgical margin, LVI, ≥pT3, and preoperative CKD (Figure 2C).

### Uni- and multivariate analyses for intravesical RFS, visceral RFS, CSS, and OS

Preoperative CKD was a significant predictor of intravesical RFS, visceral RFS, CSS, and OS (Table 2, upper row). Background-adjusted multivariate Cox regression analyses using IPTW methods demonstrated that preoperative CKD was significantly associated with poor visceral RFS (*P* = 0.003; hazard ratio [HR], 2.33, 95% confidence interval [CI], 1.34–4.04), CSS (*P* = 0.039; HR, 1.96; 95% CI, 1.03–3.70), and OS (*P* = 0.037 HR, 1.76; 95% CI, 1.04–2.99) after RNU (Table 2, lower row).

**Table 2:** Uni- and multivariate analyses for intravesical recurrence-free survival (intravesical RFS), visceral recurrence-free survival (visceral RFS), cancer-specific survival (CSS), and overall survival (OS)

Univariate	Factor	*P value*	HR	95%CI
Intravesical RFS	CKD	0.007	1.71	1.16-2.52
Visceral RFS	CKD	<0.001	3.46	2.15-5.56
CSS	CKD	<0.001	3.50	1.96-6.25
OS	CKD	<0.001	2.47	1.57-3.87

Multivariate (IPTW* methods)	Factor	*P value*	HR	95%CI
Intravesical RFS	CKD	0.145	1.40	0.89-2.18
Visceral RFS	CKD	0.003	2.33	1.34-4.04
CSS	CKD	0.039	1.96	1.03-3.70
OS	CKD	0.037	1.76	1.04-2.99

*, inverse probability of treatment weighting. Variables included in the IPTW analysis were age, sex, ECOG PS, smoking, HTN, CVD, DM, NAC, presence of hydronephrosis, tumor location, laparoscopic surgery, stage ≥pT3, pN, LVI, and surgical margin.

### The nomogram for 5-year intravesical RFS, visceral RFS, CSS probability

**Figure 3:**
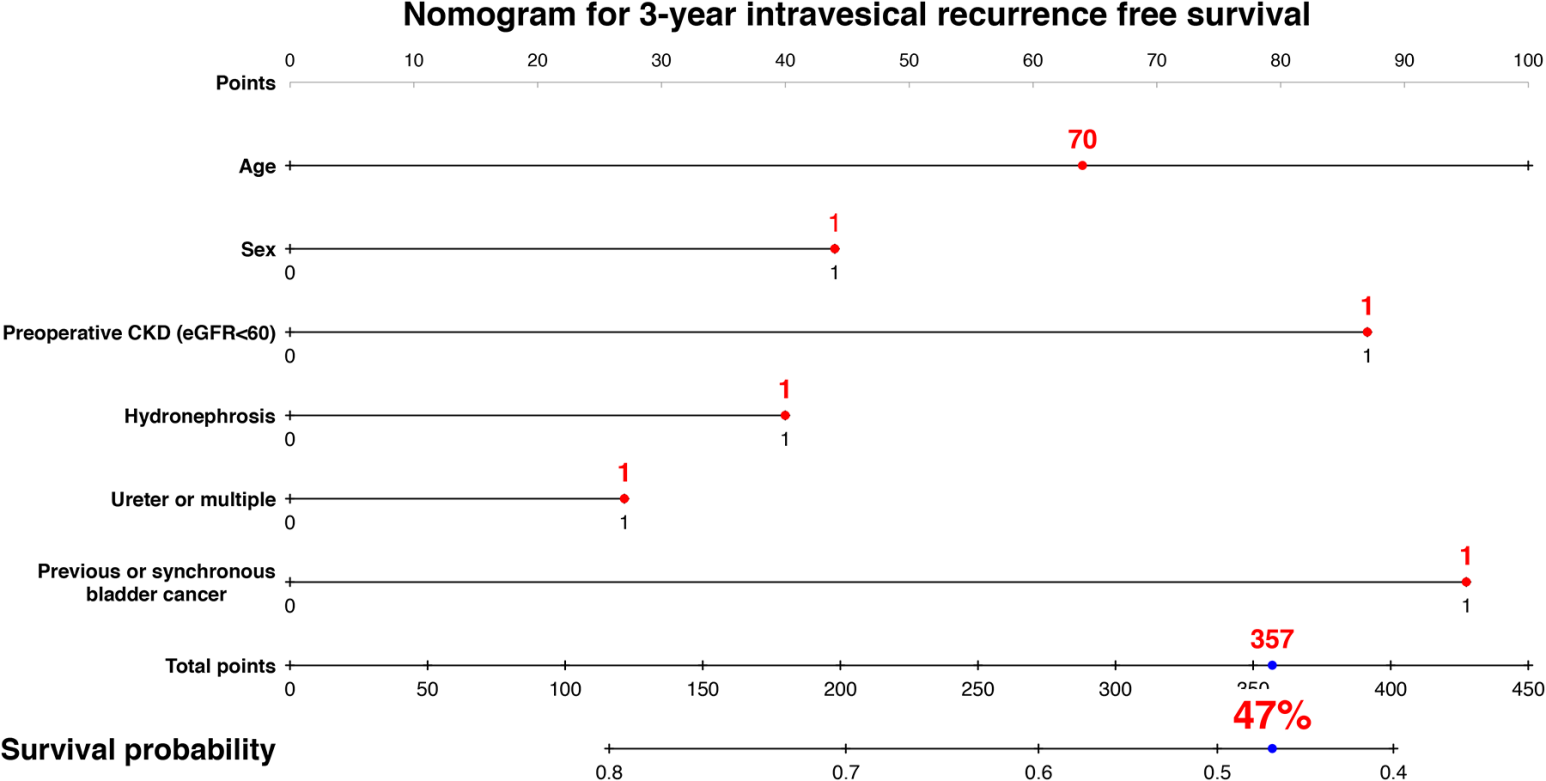
Predictive model for 3-year intravesical recurrence-free survival The nomogram including age, sex, preoperative CKD, hydronephrosis, tumor location, previous/synchronous bladder cancer (BC) for predicting 3-year intravesical recurrence-free survival is shown. The calculation for 3-year intravesical recurrence-free survival probability in the case of 70-year-old male patients who underwent radical nephroureterectomy with bladder cuff excision with preoperative CKD, hydronephrosis, ureter tumor, and positive history of previous BC provided a value of 47%. The nomogram demonstrated a significant correlation between estimated and actual intravesical recurrence-free survival (*c*-index = 0.63, *P* < 0.001, 95% CI: 0.57–0.69).

**Figure 4:**
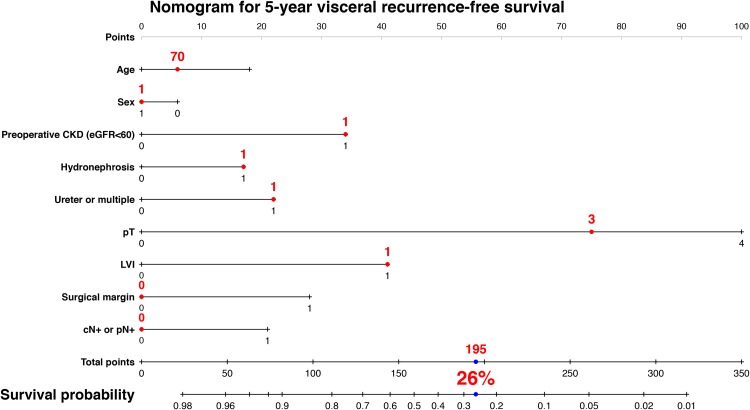
Predictive model for 5-year visceral recurrence-free survival The nomogram including age, sex, preoperative CKD, hydronephrosis, tumor location, pT, lymphovascular invasion (LVI), surgical margin, cN+ or pN+ for predicting 5-year visceral recurrence-free survival is shown. The calculation for 5-year visceral recurrence-free survival probability in the case of 70-year-old male patients who underwent radical nephroureterectomy with bladder cuff excision with preoperative CKD, hydronephrosis, ureter tumor, pT3, LVI, negative surgical margin, and negative in cN or pN provided a value of 26%. The nomogram demonstrated a significant correlation between estimated and actual visceral recurrence-free survival (*c*-index = 0.85; *P* < 0.001; 95% CI, 0.80–0.89).

**Figure 5:**
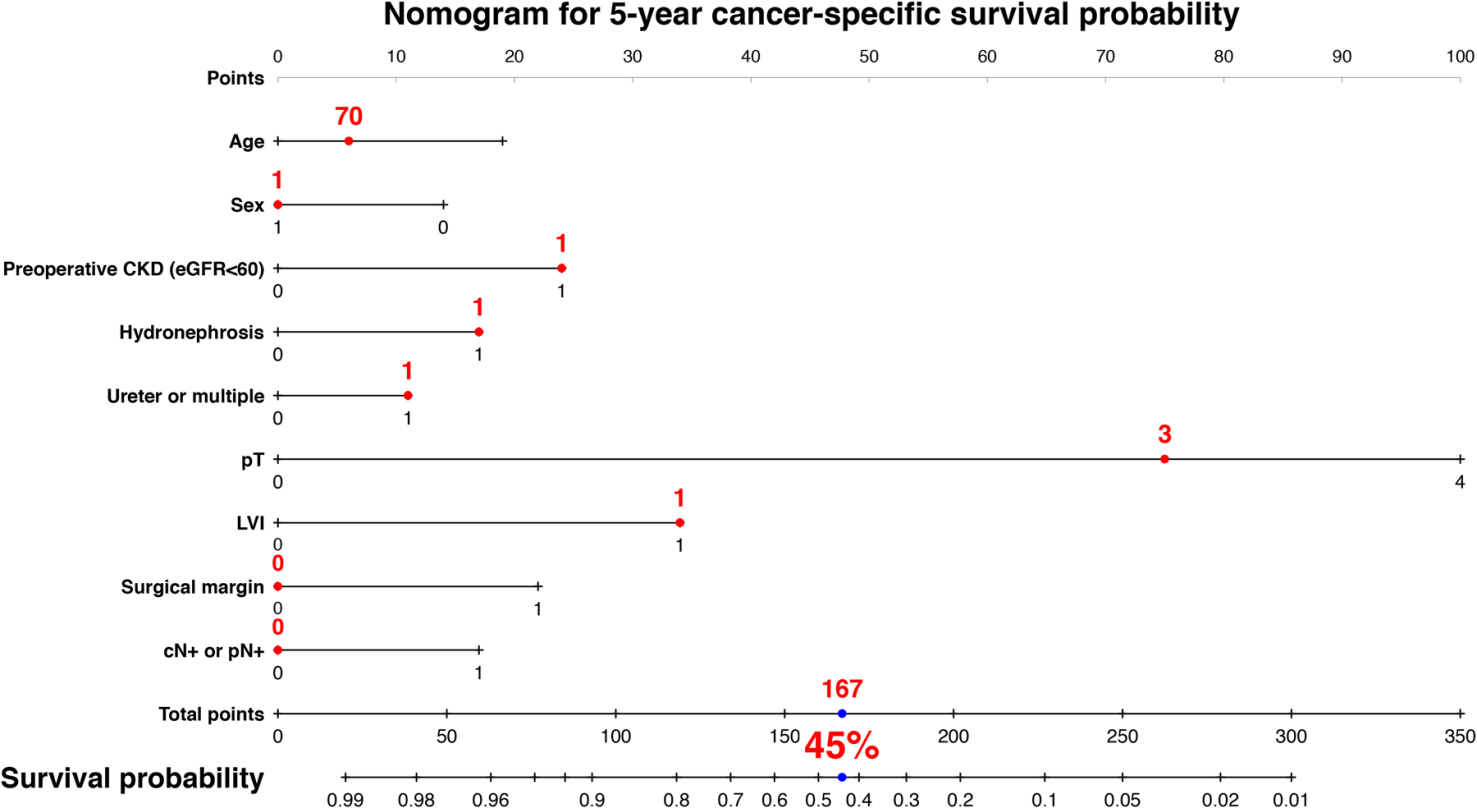
Predictive model for 5-year cancer-specific survival The nomogram including age, sex, preoperative CKD, hydronephrosis, tumor location, pT, lymphovascular invasion (LVI), surgical margin, cN+ or pN+ for predicting 5-year cancer-specific survival is shown. The calculation for 5-year visceral recurrence-free survival probability in the case of 70-year-old male patients who underwent radical nephroureterectomy with bladder cuff excision with preoperative CKD, hydronephrosis, ureter tumor, pT3, LVI, negative surgical margin, and negative in cN or pN provided a value of 45%. The nomogram demonstrated a significant correlation between estimated and actual visceral recurrence-free survival (*c*-index = 0.83; *P* < 0.001; 95% CI, 0.78–0.89).

We developed a nomogram predicting 3-year intravesical RFS including age, sex, preoperative CKD, presence of hydronephrosis, tumor location, and previous/synchronous bladder cancer (Figure 3). This model revealed a significant correlation between estimated and actual intravesical RFS (*c*-index = 0.63, *P* < 0.001, 95% CI: 0.57–0.69). To develop a nomogram predicting 5-year visceral RFS and CSS, we included age, sex, preoperative CKD, presence of hydronephrosis, pT stage, LVI, surgical margin, and cN+ or pN+. The nomogram for 5-year visceral RFS revealed a significant correlation between estimated and actual visceral RFS (Figure 4; *c*-index = 0.85; *P* < 0.001; 95% CI, 0.80–0.89). The nomogram for 5-year CSS revealed a significant correlation between estimated and actual CSS (Figure 5; *c*-index = 0.83; *P* < 0.001; 95% CI, 0.78–0.89). The risk calculations for intravesical RFS, visceral RFS, and CSS are provided in a Supplementary Files 1, 2, and 3.

## DISCUSSION

Our results demonstrated a prevalence of preoperative CKD of 59%, and found preoperative CKD to be an independent predictor of visceral RFS, CSS and OS in patients with UTUC who underwent RNU. The independent predictive value of preoperative CKD remained after multivariable Cox proportional hazards regression analysis accounting for established prognostic factors, such as ≥pT3, LVI+, and a positive surgical margin. Background-adjusted IPTW analyses identified preoperative CKD as a significant factor for prognosis. In addition, our nomogram for predicting 5-year visceral RFS and CSS demonstrated a significant correlation between estimated and actual values. As shown in Figures 4, 5 and the Supplementary File 1, preoperative CKD decreased the 5-year risk of visceral RFS from 49% to 26% (a 23% decline) in 70-year-old male patients with hydronephrosis+, ureter tumor, pT3, and LVI+. Similarly, preoperative CKD decreased the 5-year risk of CSS from 64% to 45% (a 19% decline) in the same patient population. These results suggest that the impact of preoperative CKD has important clinical implications.

As the prevalence of elderly patients is higher in UTUC, interest in the impact of preoperative renal insufficiency on prognosis has increased recently. However, a cause-and-effect relationship between renal insufficiency and malignant potential in urothelial carcinoma has yet to be established. Evidence from a prospective population-based cohort study suggested that the incidences of urinary tract malignancies increases in patients with CKD [12]. Previously, Li CE *et al.* suggested that reduced eGFR (eGFR <30 mL/min/1.73m^2^) was also associated with a higher risk of recurrence and poorer overall survival from primary bladder cancer without muscle invasion [7]. A recent meta-analysis [10] reported a prevalence of preoperative renal insufficiency among patients with bladder cancer of 16.9% (ranging from 13.0% to 25.5%) and preoperative renal insufficiency as associated with increased disease recurrence (HR = 1.65; 95% CI, 1.11–2.19), CSS (HR = 1.59; 95% CI, 1.14–2.05), and OS (HR = 1.45; 95% CI, 1.19–1.71). However, few studies have evaluated oncological outcomes according to CKD (eGFR <60 mL/min/1.73 m^2^) criteria in UTUC patients who underwent RNU [10, 11]. Therefore, our results highlight the importance of preoperative renal function, not only for neoadjuvant eligibility [4, 6, 13–15], but also for oncological outcomes after RNU [11].

On the other hand, the impact of CKD on prognosis may differ depending on cancer type. CKD is not an independent risk factor for survival in patients with lung cancer [16] or breast cancer [17, 18]. Currently, CKD is reported to be an independent risk factor for survival in head and neck, stomach, liver, colorectal, urinary tract, gynecological and hematologic malignancies [17, 19]. As genitourinary cancers are risk factors for renal dysfunction during disease progression and treatment, preoperative renal insufficiency also serves as a prognostic factor [11, 20, 21]. However, the precise biological mechanisms underlying the association between CKD, cancer type, and oncological outcomes remain poorly understood. Further studies are required to elucidate the mechanisms underlying carcinogenesis and CKD, and determine the prognostic utility of CKD in selected cancer types.

CKD is believed to be strongly associated with all-cause mortality, especially cardiovascular-related mortality [22]. However, our previous study for muscle-invasive bladder cancer indicate that CKD patients with urothelial carcinoma may develop cancers with more aggressive behaviors leading to disease progression and recurrence [21]. The reason for the strong association between CKD and cancer recurrence and mortality remains undetermined. Several studies have indicated an association between CKD and oncological outcomes related to the effects of chronic inflammation, oxidative stress, metabolic disorder, and uremia-associated immune deficiency [22–25]. Immunocompromised CKD patients may have reduced DNA repair capacity and protection against viral oncogenes [26]. Long-term inflammation and oxidative stress caused by CKD and linked to organ degradation may increase carcinogenicity. The other potential factor that can result in a poor prognosis is frailty. Patients with CKD are more likely to be frail [27] as CKD is likely to exist in combination with comorbid conditions, disability, and polypharmacy [28, 29]. In addition, frailty is also an important aspect of cancer burden [30, 31]. A recent study reported an association between frailty and inflammatory markers in elderly cancer patients [32]. Our previous study suggested that renal function has utility in predicting postoperative frailty [33]. Although we were unable to determine a definitive cause of the independent effect of CKD on UTUC recurrence, these results support a potential relationship between CKD and cancer progression.

Several limitations of the present study must be acknowledged. First, the use of data from multiple centers, the retrospective study design, and some patients with short follow-up prevented us from making definitive conclusions regarding the impact of preoperative CKD on prognosis. Despite the use of an IPTW method, which is an attractive method for estimating treatment effects using observational data, we were unable to control for selection bias and other unmeasurable confounders of retrospective studies. Second, eGFR evaluation using a modified formula for Japanese patients may prevent the generalization of our results to non-Asian populations. Third, a separate validation cohort is required to verify the accuracy of our nomograms. In addition, a prospective study of the relationship between preoperative CKD and oncological outcomes is necessary. Despite these limitations, we evaluated the direct impact of CKD on oncological outcomes in UTUC patients using IPTW analysis and developed a prognostic factor-based risk stratification nomogram. Because no prospective study is available to compare the influence of impaired renal function on prognosis, our results support the rationale that preoperative CKD is an important predictor of cancer mortality in patients with UTUC.

In conclusion, UTUC patients with preoperative CKD had significantly lower survival than those without CKD after RNU. Further studies are required to assess the impact of renal insufficiency on the prognosis of UTUC.

## MATERIALS AND METHODS

### Design and ethics statement

The present retrospective, multicenter study was performed in accordance with the ethical standards of the Declaration of Helsinki and approved by an ethics review board of Hirosaki University School of Medicine (authorization numbers; 2015–258 and 2016–225).

### Patient selection

Between February 1995 and February 2017, 426 adults underwent RNU with bladder cuff excision in Hirosaki University Hospital, Aomori Rosai Hospital, Mutsu General Hospital, Tsugaru General Hospital, and Aomori Prefectural Central Hospital. We stratified patients into two groups according to preoperative renal function as follows: eGFR ≥60 mL/min/1.73 m^2^ (non-CKD group) and eGFR <60 mL/min/1.73 m^2^ (CKD group).

### Evaluation of variables

The variables analyzed were age, sex, Eastern Cooperative Oncology Group performance status (ECOG PS), smoking, clinical stage, renal function before RNU, history of hypertension (HTN), cardiovascular disease (CVD), and diabetes mellitus (DM). Renal function was evaluated by eGFR before RNU using a modified version of the abbreviated Modification of Diet in Renal Disease Study formula for Japanese patients [34]. CKD was defined as a preoperative eGFR < 60 mL/min/1.73m^2^. Tumor stage and grade were assigned according to the 2009 TNM classification of the Union of International Cancer Control [35]. Postoperative complications were evaluated using the Clavien–Dindo classification [36].

### Neoadjuvant chemotherapy (NAC)

Since September 2006, we have performed two to four courses of NAC for the treatment of locally advanced UTUC (cT3-4 and/or cN+) in selected patients. NAC comprised a platinum-based combination regimen using either gemcitabine plus cisplatin; gemcitabine plus carboplatin; or methotrexate, vinblastine, adriamycin, and cisplatin. Regimens were selected based on guidelines regarding eligibility for the proper use of cisplatin [37] and the patient’s overall status.

### Surgical procedure

Open or laparoscopic nephroureterectomy, which includes the removal of kidney, ureter, and ipsilateral bladder cuff, was performed [2]. The distal ureter was managed by the extravesical approach. A regional lymph node dissection was performed depending on tumor stage. We did not use early (within 48 h) intravesical chemotherapy after nephroureterectomy.

### Patient follow-up

Oncological follow-up after RNU was performed according to the European Association of Urology guidelines [1] and the Japanese guidelines for UTUC [5] and bladder cancer [38]. Our follow-up protocol consisted of complete blood counts, serum chemistry screenings, urine cytology, cystoscopy, ultrasound imaging of abdomen, computed tomography (CT), and chest radiography every 3–6 months (based on pathologic findings) for at least five years. Adjuvant chemotherapy was not administered routinely. Salvage therapy was introduced when indicated by CT.

### Outcome evaluations

We evaluated pathological T and N stages, LVI, and surgical margin in the non-CKD and CKD groups. Oncological outcomes for both groups, including intravesical RFS, visceral RFS, CSS, and OS, were investigated using the Kaplan–Meier method and compared with the log-rank test. Multivariate Cox regression analysis was performed for independent predictors of intravesical RFS, visceral RFS, CSS, and OS.

### Statistical analysis

Statistical analyses of data were performed using SPSS version 24.0 (SPSS, Inc., Chicago, IL, USA), GraphPad Prism 5.03 (GraphPad Software, San Diego, CA, USA), and R 3.3.2 (The R Foundation for Statistical Computing, Vienna, Austria). Categorical variables were compared using Fisher’s exact test or the χ^2^ test. Quantitative variables were expressed as mean with standard deviation (SD) or median with interquartile range. Differences between groups were compared statistically using Student’s *t*-test for data with a normal distribution or the Mann–Whitney *U* test for data with a non-normal distribution. *P* values < 0.05 were considered statistically significant.

Cox proportional hazards regression models were used to evaluate the impact of CKD on prognosis. HRs with 95% CIs were calculated after controlling for potential confounders, including patient demographics and clinicopathologic tumor variables. Additionally, we performed a Cox proportional hazards regression analysis using IPTW, which performs reweighting of affected and unaffected groups to emulate a propensity score-matched population [39] in order to evaluate the impact of preoperative CKD on prognosis. Variables included in the IPTW analysis were age, sex, ECOG PS, smoking, HTN, CVD, DM, NAC, presence of hydronephrosis, tumor location, laparoscopic surgery, stage ≥pT3, pN, LVI, and surgical margin. We developed a prognostic factor-based risk stratification nomogram for 3-year intravesical RFS, 5-year visceral RFS, and 5-year CSS with Cox proportional hazards regression analyses using the “rms” library in R. The *c*-index for predicting overall survival probability was calculated as the area under the receiver operating characteristic curve.

## SUPPLEMENTARY MATERIALS



## Abbreviations

CKD: chronic kidney disease
IPTW: inverse probability of treatment weighting
RNU: radical nephroureterectomy
RFS: recurrence-free survival
CSS: cancer-specific survival
OS: overall survival
UTUC: upper tract urothelial carcinoma
LVI: lymphovascular invasion
eGFR: estimated glomerular filtration rate
HR: hazard ratio
CI: confidence interval
ECOG PS: Eastern Cooperative Oncology Group performance status
HTN: hypertension
CVD: cardiovascular disease
DM: diabetes mellitus
NAC: neoadjuvant chemotherapy
CT: computed tomography
SD: standard deviation
CIS: carcinoma *in situ*
