# Supplementary material for: Preoperative chronic kidney disease predicts poor oncological outcomes after radical nephroureterectomy in patients with upper urinary tract urothelial carcinoma
Source: Oncotarget. 2017 Aug 24;8(47):83183–94. doi: 10.18632/oncotarget.20554 (PMC5669959; doi:10.18632/oncotarget.20554)
Supplement: Supplementary file 1 [file oncotarget-08-83183-s001.pdf]

Preoperative chronic kidney disease predicts poor oncological outcomes after radical nephroureterectomy in patients with upper urinary tract urothelial carcinoma

SUPPLEMENTARY MATERIALS

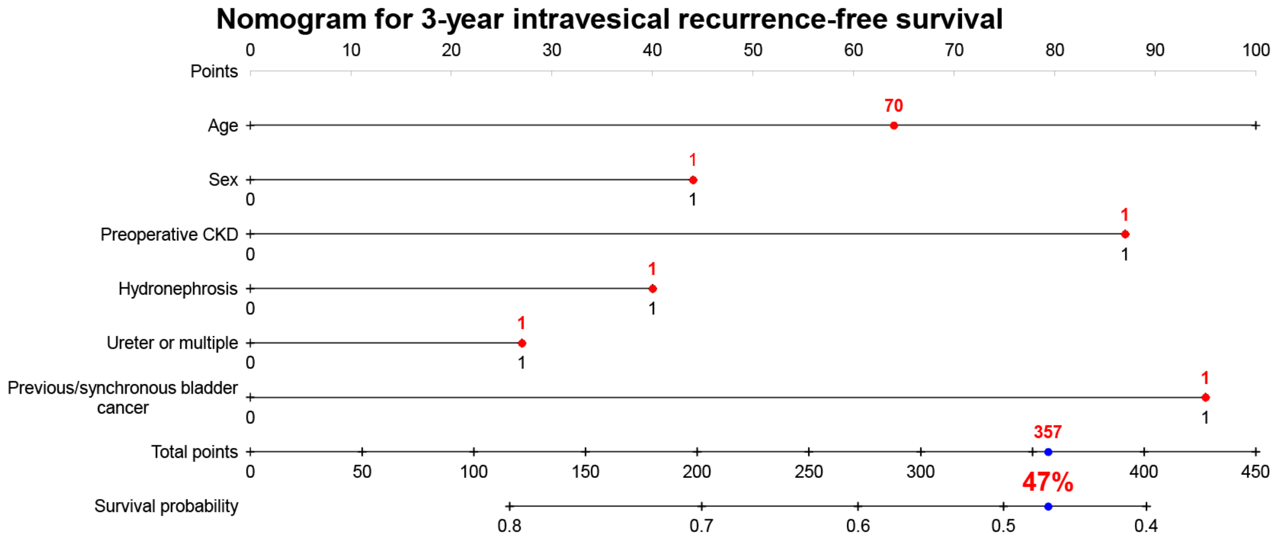

Supplementary File 1: Nomogram for intravesical recurrence-free survival.

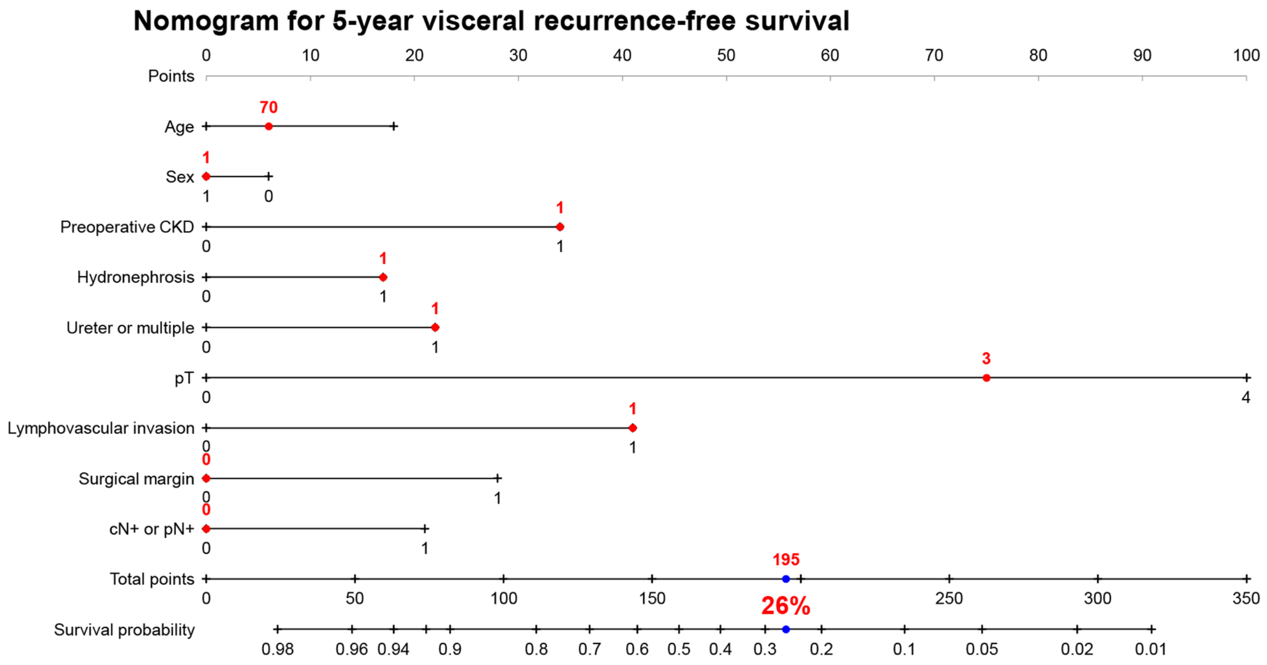

Supplementary File 2: Nomogram for visceral recurrence-free survival.

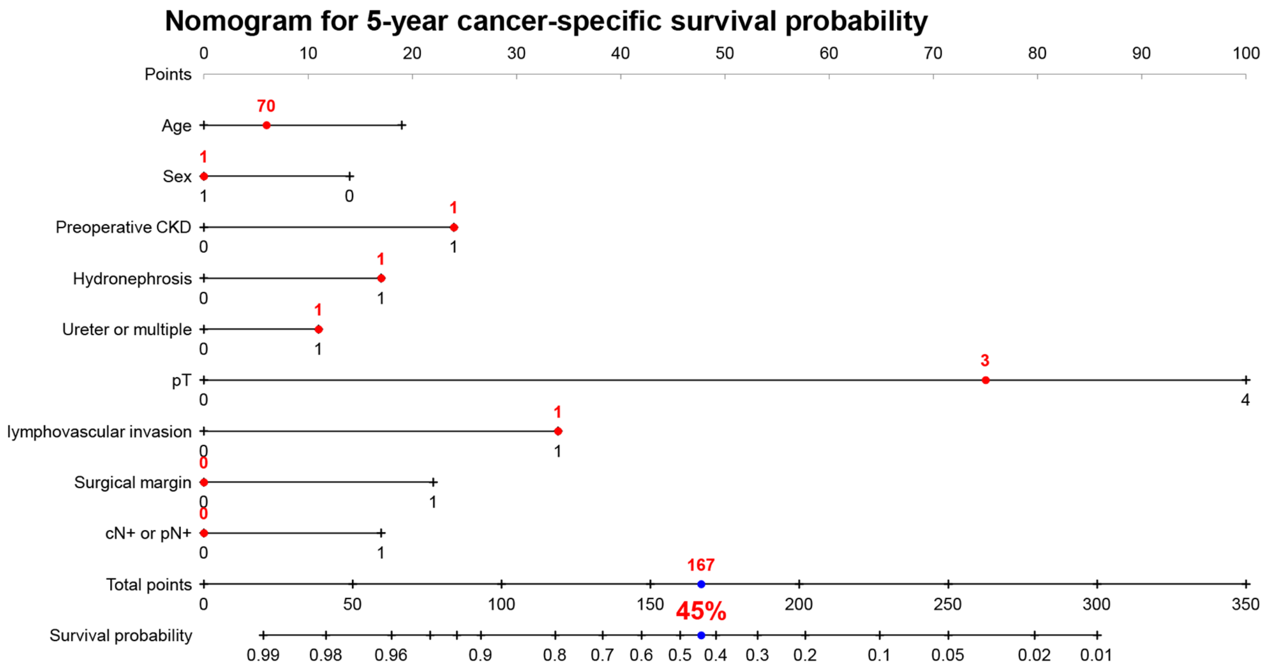

Supplementary File 3: Nomogram for cancer-specific survival.
